# (*E*)-*tert*-Butyl 4-(*N*′-hy­droxy­carbam­­imid­o­yl)piperazine-1-carboxyl­ate

**DOI:** 10.1107/S1600536812046223

**Published:** 2012-11-14

**Authors:** S. Sreenivasa, K. E. Manojkumar, P. A. Suchetan, N. R. Mohan, B. S. Palakshamurthy

**Affiliations:** a Department of Studies and Research in Chemistry, Tumkur University, Tumkur, Karnataka 572 103, India; b Department of Studies and Research in Chemistry, U.C.S, Tumkur University, Tumkur, Karnataka 572 103, India; c Department of Studies and Research in Physics, U.C.S, Tumkur University, Tumkur, Karnataka 572 103, India

## Abstract

In the title compound, C_10_H_20_N_4_O_3_, the piperazine ring adopts a chair conformation. The mol­ecule adopts an *E* conformation across the C=N double bond, with the –OH group and the piperazine ring *trans* to one another. Further, the H atom of the hy­droxy group is directed away from the NH_2_ group. An intra­molecular N—H⋯O contact occurs involving the NH_2_ group and the oxime O atom. In the crystal, mol­ecules are linked *via* strong N—H⋯O and O—H⋯N hydrogen bonds with alternating *R*_2_^2^(6) and *C*(9) motifs into tetra­meric units forming *R*_4_^4^(28) motifs.

## Related literature

For the synthesis, characterization and biological activity of piperazine and its derivatives, see: Gan *et al.* (2009*a* ▶,*b* ▶); Willems & Ilzerman (2010 ▶). For a related structure, see: Gowda *et al.* (2009 ▶). For hydrogen-bond motifs, see: Bernstein *et al.* (1995 ▶); Etter (1990 ▶).
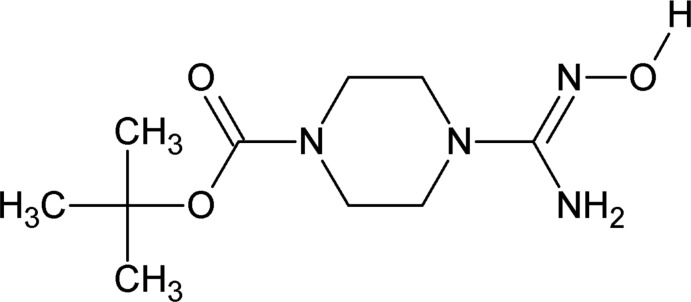


## Experimental

### 

#### Crystal data

C_10_H_20_N_4_O_3_
*M* *_r_* = 244.3
Triclinic, 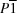

*a* = 8.1923 (17) Å
*b* = 8.7859 (16) Å
*c* = 9.714 (2) Å
α = 109.451 (7)°
β = 99.540 (7)°
γ = 96.474 (7)°
*V* = 639.5 (2) Å^3^
*Z* = 2
Mo *K*α radiation
μ = 0.10 mm^−1^
*T* = 300 K
0.22 × 0.16 × 0.1 mm

#### Data collection

Bruker SMART X2S diffractometer
6407 measured reflections
2218 independent reflections
1632 reflections with *I* > 2σ(*I*)
*R*_int_ = 0.027

#### Refinement

*R*[*F* ^2^ > 2σ(*F* ^2^)] = 0.044
*wR*(*F* ^2^) = 0.130
*S* = 1.05
2218 reflections
162 parameters
H atoms treated by a mixture of independent and constrained refinement
Δρ_max_ = 0.21 e Å^−3^
Δρ_min_ = −0.20 e Å^−3^

### 

Data collection: *SMART* (Bruker, 2004 ▶); cell refinement: *SAINT-Plus* (Bruker, 2004 ▶); data reduction: *SAINT-Plus* and *XPREP* (Bruker, 2004 ▶); program(s) used to solve structure: *SHELXS97* (Sheldrick, 2008 ▶); program(s) used to refine structure: *SHELXL97* (Sheldrick, 2008 ▶); molecular graphics: *ORTEP-3* (Farrugia, 2012 ▶); software used to prepare material for publication: *SHELXL97*.

## Supplementary Material

Crystal structure: contains datablock(s) I, global. DOI: 10.1107/S1600536812046223/zj2096sup1.cif

Structure factors: contains datablock(s) I. DOI: 10.1107/S1600536812046223/zj2096Isup2.hkl

Supplementary material file. DOI: 10.1107/S1600536812046223/zj2096Isup3.cml

Additional supplementary materials:  crystallographic information; 3D view; checkCIF report

## Figures and Tables

**Table 1:** Hydrogen-bond geometry (Å, °)

*D*—H⋯*A*	*D*—H	H⋯*A*	*D*⋯*A*	*D*—H⋯*A*
N2—H2*D*⋯O1	0.94 (3)	2.08 (3)	2.538 (2)	108.3 (19)
N2—H2*C*⋯O2^i^	0.89 (3)	2.10 (3)	2.988 (2)	173 (3)
O1—H1⋯N1^ii^	0.82	2.04	2.764 (3)	147

Symmetry codes: (i) 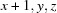; (ii) 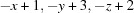.

**Fig. 1.**
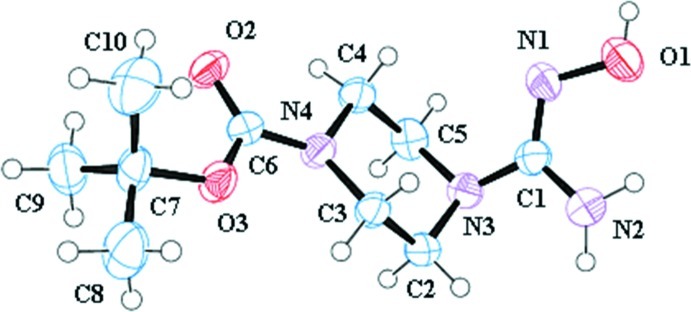
Molecular Structure of the title compound, Showing the atom-labled Scheme.

**Fig. 2.**
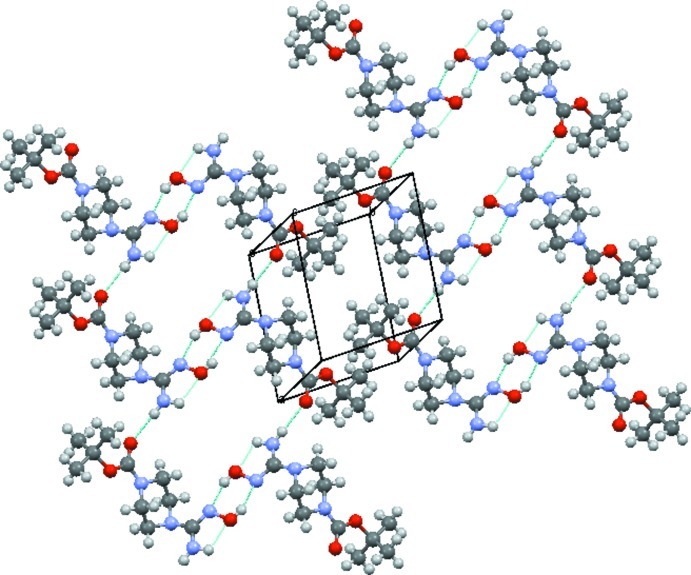
Molecular packing of the title compound, Hydrogen bonds are shown in dashed lines.

## supplementary crystallographic information

### Comment

Numerous piperazine derivatives like aryl amide, sulphonamides, Mannich bases, Schiff's bases, thiazolidinones, azetidinones, imidazolinones have shown a wide spectrum of biological activities *viz*. antiinflammatory, antibacterial, antimalarial, anticonvulsant, antipyretic, antitumor, anthelmintics, analgesic, antidepressant, antifungal, antitubercular, anticancer, antidiabetic *etc*. In this view, we synthesized the title compound to study its crystal structure. The molecule crystallizes in triclinic P-1 space group. The piperazine ring in the molecule adopts chair conformation and the molecule prefers E configuration across the C—N double bond, as the OH group and the piperazine ring are in the opposite side of the double bond (Figure 1). The hydrogen atom of the hydroxyl group is directed away from the NH_2_ group. This results in stabilizing the structure through a strong intermolecular O—H···N(1) and an intramolecular N(2)—H···O hydrogen bonds. In addition to this, the molecule also exhibits a strong N(2)—H···O(C) intermolecular hydrogen bond. The molecules are connected through alternate *R*_2_^2^(6) ring and C(9) chain hydrogen bond patterns into tetrameric units exhibiting *R*_4_^4^(28) ring patterns (Figure 2). The average N—C bond length in the piperazine ring is 1.466 Å indicating the single bond nature. While, the N4—C(O) bond length is 1.359 (2) Å indicating the delocalization of the nitrogen lone pair of electrons into π system of the carbonyl group. The N(1)—C(1) bond length is 1.290 Å due to its double bond nature, but the N(3)—C(1) and N(2)—C(1) bond lengths are closer to N—C(O) lengths indicating the partial double bond nature of these bonds.

### Experimental

To a solution of N-boc-piperazine (10.6 mmol) in 20 ml of acetonitrile was added cyanogen bromide (10.7 mmol) and K_2_CO_3_ (21.2 mmol) at -10°C. The reaction mixture was stirred for 18 h at room temperature under nitrogen atmosphere. *N*-Cyano-4-boc-piperazine was obtained. To *N*-cyano-4-boc-piperazine (4.6 mmol) in methanol was added NH_2_OH.HCl (9.3 mmol) and stirred for 30 min at room temperature. The solvent was removed under reduced pressure and the crude product was washed with cold water and dried to yield white solid product. Single crystals employed in X-ray diffraction studies were obtained from slow evaporation of the solution of the compound in methanol.

### Refinement

The H atoms were positioned with idealized geometry using a riding model with C—H = 0.93 Å. All H atoms were refined with isotropic displacement parameters (set to 1.2 times of the *U*_eq_ of the parent atom).

### Crystal data

**Table:**

C_10_H_20_N_4_O_3_	*F*(000) = 264
*M**_r_* = 244.3	prism
Triclinic, *P*1	*D*_x_ = 1.269 Mg m^−^^3^
Hall symbol: -P 1	Melting point: 463 K
*a* = 8.1923 (17) Å	Mo *K*α radiation, λ = 0.71073 Å
*b* = 8.7859 (16) Å	Cell parameters from 1632 reflections
*c* = 9.714 (2) Å	θ = 25°
α = 109.451 (7)°	µ = 0.10 mm^−^^1^
β = 99.540 (7)°	*T* = 300 K
γ = 96.474 (7)°	Prism, colorless
*V* = 639.5 (2) Å^3^	0.22 × 0.16 × 0.1 mm
*Z* = 2	

### Data collection

**Table:**

Bruker SMART X2S diffractometer	1632 reflections with *I* > 2σ(*I*)
Radiation source: fine-focus sealed tube	*R*_int_ = 0.027
Graphite monochromator	θ_max_ = 25.0°, θ_min_ = 2.5°
multi–scan	*h* = −9→9
6407 measured reflections	*k* = −10→10
2218 independent reflections	*l* = −11→11

### Refinement

**Table:**

Refinement on *F*^2^	Primary atom site location: structure-invariant direct methods
Least-squares matrix: full	Secondary atom site location: difference Fourier map
*R*[*F*^2^ > 2σ(*F*^2^)] = 0.044	Hydrogen site location: inferred from neighbouring sites
*wR*(*F*^2^) = 0.130	H atoms treated by a mixture of independent and constrained refinement
*S* = 1.05	*w* = 1/[σ^2^(*F*_o_^2^) + (0.0714*P*)^2^ + 0.0663*P*] where *P* = (*F*_o_^2^ + 2*F*_c_^2^)/3
2218 reflections	(Δ/σ)_max_ < 0.001
162 parameters	Δρ_max_ = 0.21 e Å^−^^3^
0 restraints	Δρ_min_ = −0.20 e Å^−^^3^
0 constraints	

### Special details

**Table:**

Geometry. All e.s.d.'s (except the e.s.d. in the dihedral angle between two l.s. planes) are estimated using the full covariance matrix. The cell e.s.d.'s are taken into account individually in the estimation of e.s.d.'s in distances, angles and torsion angles; correlations between e.s.d.'s in cell parameters are only used when they are defined by crystal symmetry. An approximate (isotropic) treatment of cell e.s.d.'s is used for estimating e.s.d.'s involving l.s. planes.
Refinement. Refinement of *F*^2^ against ALL reflections. The weighted *R*-factor *wR* and goodness of fit *S* are based on *F*^2^, conventional *R*-factors *R* are based on *F*, with *F* set to zero for negative *F*^2^. The threshold expression of *F*^2^ > σ(*F*^2^) is used only for calculating *R*-factors(gt) *etc*. and is not relevant to the choice of reflections for refinement. *R*-factors based on *F*^2^ are statistically about twice as large as those based on *F*, and *R*- factors based on ALL data will be even larger.

### Fractional atomic coordinates and isotropic or equivalent isotropic displacement parameters (Å^2^)

**Table:**

	*x*	*y*	*z*	*U*_iso_*/*U*_eq_	
C8	−0.0609 (3)	0.4383 (3)	0.7235 (3)	0.0705 (7)	
H8A	−0.1504	0.3592	0.7258	0.106*	
H8B	0.0275	0.4655	0.8107	0.106*	
H8C	−0.0177	0.3931	0.6354	0.106*	
C7	−0.1275 (2)	0.5921 (2)	0.7215 (2)	0.0452 (5)	
O3	0.02502 (15)	0.69800 (15)	0.71797 (16)	0.0449 (4)	
C6	0.0186 (2)	0.8470 (2)	0.7099 (2)	0.0363 (4)	
N4	0.17149 (18)	0.92339 (18)	0.70828 (17)	0.0379 (4)	
C3	0.3293 (2)	0.8718 (2)	0.7533 (2)	0.0386 (5)	
H3A	0.3094	0.7539	0.7291	0.046*	
H3B	0.3705	0.9241	0.8606	0.046*	
C2	0.4601 (2)	0.9178 (2)	0.6738 (2)	0.0426 (5)	
H2A	0.5652	0.8871	0.7081	0.051*	
H2B	0.423	0.8574	0.5672	0.051*	
N3	0.48827 (18)	1.09515 (18)	0.70205 (16)	0.0380 (4)	
C1	0.5834 (2)	1.1978 (2)	0.8426 (2)	0.0372 (5)	
C10	−0.1871 (3)	0.6755 (3)	0.8630 (3)	0.0710 (7)	
H10A	−0.2858	0.6076	0.866	0.106*	
H10B	−0.2133	0.7796	0.864	0.106*	
H10C	−0.0999	0.6921	0.9486	0.106*	
O2	−0.10816 (16)	0.90611 (16)	0.70148 (16)	0.0489 (4)	
N2	0.7249 (2)	1.1515 (2)	0.8972 (2)	0.0500 (5)	
C5	0.3302 (2)	1.1390 (2)	0.6480 (2)	0.0437 (5)	
H5A	0.2963	1.083	0.5405	0.052*	
H5B	0.3481	1.2561	0.6682	0.052*	
C4	0.1884 (2)	1.0956 (2)	0.7193 (2)	0.0422 (5)	
H4A	0.2114	1.1666	0.8236	0.051*	
H4B	0.0835	1.1134	0.6693	0.051*	
C9	−0.2609 (3)	0.5532 (3)	0.5819 (3)	0.0623 (6)	
H9A	−0.3586	0.4846	0.5859	0.093*	
H9B	−0.2185	0.4968	0.496	0.093*	
H9C	−0.2907	0.6533	0.5747	0.093*	
O1	0.6559 (2)	1.42316 (18)	1.05009 (17)	0.0655 (5)	
H1	0.6236	1.5071	1.0951	0.098*	
N1	0.5327 (2)	1.33013 (19)	0.91361 (19)	0.0483 (5)	
H2C	0.776 (3)	1.084 (3)	0.835 (3)	0.061 (7)*	
H2D	0.789 (3)	1.232 (3)	0.986 (3)	0.076 (8)*	

### Atomic displacement parameters (Å^2^)

**Table:**

	*U*^11^	*U*^22^	*U*^33^	*U*^12^	*U*^13^	*U*^23^
C8	0.0538 (14)	0.0579 (14)	0.113 (2)	0.0029 (12)	0.0172 (14)	0.0500 (15)
C7	0.0329 (10)	0.0476 (12)	0.0584 (13)	−0.0013 (9)	0.0116 (9)	0.0252 (10)
O3	0.0339 (7)	0.0396 (8)	0.0685 (9)	0.0052 (6)	0.0167 (7)	0.0264 (7)
C6	0.0348 (10)	0.0372 (10)	0.0377 (10)	0.0091 (8)	0.0113 (8)	0.0119 (8)
N4	0.0307 (8)	0.0338 (8)	0.0539 (10)	0.0079 (6)	0.0134 (7)	0.0191 (7)
C3	0.0327 (10)	0.0316 (10)	0.0541 (12)	0.0074 (8)	0.0106 (9)	0.0176 (9)
C2	0.0378 (11)	0.0360 (10)	0.0500 (12)	0.0067 (8)	0.0149 (9)	0.0077 (9)
N3	0.0399 (9)	0.0358 (9)	0.0411 (9)	0.0045 (7)	0.0144 (7)	0.0153 (7)
C1	0.0357 (10)	0.0344 (10)	0.0457 (11)	0.0046 (8)	0.0150 (9)	0.0173 (9)
C10	0.0646 (15)	0.0950 (19)	0.0633 (15)	0.0070 (14)	0.0278 (13)	0.0369 (14)
O2	0.0332 (8)	0.0476 (8)	0.0696 (10)	0.0143 (6)	0.0153 (7)	0.0214 (7)
N2	0.0422 (10)	0.0483 (11)	0.0554 (12)	0.0131 (9)	0.0093 (9)	0.0125 (9)
C5	0.0481 (12)	0.0424 (11)	0.0436 (11)	0.0037 (9)	0.0060 (9)	0.0225 (9)
C4	0.0390 (11)	0.0326 (10)	0.0571 (12)	0.0106 (8)	0.0091 (9)	0.0182 (9)
C9	0.0512 (13)	0.0536 (13)	0.0695 (16)	−0.0039 (11)	0.0003 (12)	0.0164 (12)
O1	0.0646 (10)	0.0469 (9)	0.0624 (10)	0.0096 (8)	−0.0060 (8)	0.0004 (7)
N1	0.0436 (10)	0.0355 (9)	0.0547 (11)	0.0041 (8)	0.0012 (8)	0.0073 (8)

### Geometric parameters (Å, º)

**Table:**

C8—C7	1.517 (3)	N3—C5	1.457 (2)
C8—H8A	0.96	C1—N1	1.292 (2)
C8—H8B	0.96	C1—N2	1.356 (3)
C8—H8C	0.96	C10—H10A	0.96
C7—O3	1.484 (2)	C10—H10B	0.96
C7—C9	1.506 (3)	C10—H10C	0.96
C7—C10	1.515 (3)	N2—H2C	0.89 (3)
O3—C6	1.343 (2)	N2—H2D	0.94 (3)
C6—O2	1.216 (2)	C5—C4	1.522 (3)
C6—N4	1.358 (2)	C5—H5A	0.97
N4—C3	1.465 (2)	C5—H5B	0.97
N4—C4	1.470 (2)	C4—H4A	0.97
C3—C2	1.514 (3)	C4—H4B	0.97
C3—H3A	0.97	C9—H9A	0.96
C3—H3B	0.97	C9—H9B	0.96
C2—N3	1.473 (2)	C9—H9C	0.96
C2—H2A	0.97	O1—N1	1.447 (2)
C2—H2B	0.97	O1—H1	0.82
N3—C1	1.398 (2)		
			
C7—C8—H8A	109.5	C5—N3—C2	108.79 (15)
C7—C8—H8B	109.5	N1—C1—N2	123.51 (19)
H8A—C8—H8B	109.5	N1—C1—N3	119.03 (17)
C7—C8—H8C	109.5	N2—C1—N3	117.45 (17)
H8A—C8—H8C	109.5	C7—C10—H10A	109.5
H8B—C8—H8C	109.5	C7—C10—H10B	109.5
O3—C7—C9	110.52 (16)	H10A—C10—H10B	109.5
O3—C7—C10	109.00 (17)	C7—C10—H10C	109.5
C9—C7—C10	112.76 (19)	H10A—C10—H10C	109.5
O3—C7—C8	101.95 (15)	H10B—C10—H10C	109.5
C9—C7—C8	110.19 (18)	C1—N2—H2C	119.9 (15)
C10—C7—C8	111.90 (18)	C1—N2—H2D	112.7 (15)
C6—O3—C7	121.19 (14)	H2C—N2—H2D	120 (2)
O2—C6—O3	124.82 (16)	N3—C5—C4	113.42 (14)
O2—C6—N4	123.46 (17)	N3—C5—H5A	108.9
O3—C6—N4	111.70 (15)	C4—C5—H5A	108.9
C6—N4—C3	123.10 (15)	N3—C5—H5B	108.9
C6—N4—C4	117.64 (15)	C4—C5—H5B	108.9
C3—N4—C4	115.06 (15)	H5A—C5—H5B	107.7
N4—C3—C2	110.37 (15)	N4—C4—C5	110.69 (15)
N4—C3—H3A	109.6	N4—C4—H4A	109.5
C2—C3—H3A	109.6	C5—C4—H4A	109.5
N4—C3—H3B	109.6	N4—C4—H4B	109.5
C2—C3—H3B	109.6	C5—C4—H4B	109.5
H3A—C3—H3B	108.1	H4A—C4—H4B	108.1
N3—C2—C3	111.33 (14)	C7—C9—H9A	109.5
N3—C2—H2A	109.4	C7—C9—H9B	109.5
C3—C2—H2A	109.4	H9A—C9—H9B	109.5
N3—C2—H2B	109.4	C7—C9—H9C	109.5
C3—C2—H2B	109.4	H9A—C9—H9C	109.5
H2A—C2—H2B	108	H9B—C9—H9C	109.5
C1—N3—C5	117.38 (15)	N1—O1—H1	109.5
C1—N3—C2	116.57 (15)	C1—N1—O1	109.58 (16)
			
C9—C7—O3—C6	60.5 (2)	C3—C2—N3—C5	−59.90 (19)
C10—C7—O3—C6	−64.0 (2)	C5—N3—C1—N1	−3.9 (2)
C8—C7—O3—C6	177.58 (17)	C2—N3—C1—N1	−135.54 (18)
C7—O3—C6—O2	−1.7 (3)	C5—N3—C1—N2	175.79 (15)
C7—O3—C6—N4	179.86 (15)	C2—N3—C1—N2	44.1 (2)
O2—C6—N4—C3	165.07 (18)	C1—N3—C5—C4	−77.5 (2)
O3—C6—N4—C3	−16.5 (2)	C2—N3—C5—C4	57.6 (2)
O2—C6—N4—C4	9.3 (3)	C6—N4—C4—C5	−154.43 (16)
O3—C6—N4—C4	−172.28 (15)	C3—N4—C4—C5	47.9 (2)
C6—N4—C3—C2	152.67 (16)	N3—C5—C4—N4	−51.3 (2)
C4—N4—C3—C2	−51.0 (2)	N2—C1—N1—O1	4.7 (3)
N4—C3—C2—N3	56.6 (2)	N3—C1—N1—O1	−175.65 (15)
C3—C2—N3—C1	75.6 (2)		

### Hydrogen-bond geometry (Å, º)

**Table:**

*D*—H···*A*	*D*—H	H···*A*	*D*···*A*	*D*—H···*A*
N2—H2*D*···O1	0.94 (3)	2.08 (3)	2.538 (2)	108.3 (19)
N2—H2*C*···O2^i^	0.89 (3)	2.10 (3)	2.988 (2)	173 (3)
O1—H1···N1^ii^	0.82	2.04	2.764 (3)	147

Symmetry codes: (i) *x*+1, *y*, *z*; (ii) −*x*+1, −*y*+3, −*z*+2.
